# Biodegradation of Poly (Butylene Succinate) (PBS)/Stearate Modified Magnesium-Aluminium Layered Double Hydroxide Composites under Marine Conditions Prepared via Melt Compounding

**DOI:** 10.3390/molecules25235766

**Published:** 2020-12-07

**Authors:** Parameswaran Shaiju, Benamor-Bois Dorian, Ramsankar Senthamaraikannan, Ramesh Babu Padamati

**Affiliations:** 1 BiOrbic, Bioeconomy Research Centre, University College Dublin, Belfield, D04 V1W8 Dublin, Ireland; sparames@tcd.ie; 2 AMBER Centre, CRANN Institute, School of Physics, Trinity College Dublin, D02 PN40 Dublin, Ireland; d.benamorbois@gmail.com (B.-B.D.); ramsankar.s@tcd.ie (R.S.)

**Keywords:** polybutylene succinate, layered double hydroxide, composites, biodegradation, marine conditions

## Abstract

In the present work, polybutylene succinate (PBS)/stearate modified magnesium-aluminium layered double hydroxide (St-Mg-Al LDH) composites were prepared via melt processing and the effect of different loadings of St-Mg-Al LDH on the degradation behaviour of PBS under marine conditions was investigated. The morphological, mechanical and thermal characteristics of the composites were studied using different characterisation techniques. Optical imaging and scanning electron microscopy revealed that the incorporation of St-Mg-Al LDH accelerates the degradation of PBS along with the activity of microorganisms adhered to the composite films. PBS/St-Mg-Al LDH composites are found to have lower thermal degradation temperatures than those of pure PBS. The decrease in thermal stability is correlated with the degradation of PBS due to the catalytic action Mg and Al present in LDH. Tensile and DMA analysis revealed that the addition of St-Mg-Al LDH did not have a significant impact on the mechanical properties of PBS.

## 1. Introduction

Globally, an estimated 10 million tons of plastic waste enter into terrestrial and marine environments every year. The accumulation of plastic waste in the ocean creates grave environmental concerns due to their persistence and effects on marine wildlife and, potentially, humans [1]. Plastics are durable and do not biodegrade entirely in the marine environment; instead, they break up into microplastics and enter into marine life and the human food chain. In this regard, biodegradable polymers are getting enormous attention due to their ability to address the environmental issues created by the overuse and mismanagement of petroleum-based polymers. Biodegradation, a process in which the polymer chain is cleaved by the action of enzymes secreted by bacteria or fungi [2,3], is a promising method to tackle the issues associated with plastic pollution [4].

Among various biodegradable polymers, polybutylene succinate (PBS) is considered as a promising alternative to the traditional non-degradable polymers due to its comparable mechanical properties and biodegradability under multiple environments [5,6,7]. PBS can be produced either from renewable or petroleum resources [6,8]. PBS has got a wide range of applications such as packaging films, agriculture mulch films, packaging materials, vegetation nets, compost bags and other commodity applications [7,9]. However, the poor tensile properties, low melt viscosity and gas barrier properties are some of the major drawbacks which limit the applications of PBS [10,11]. Incorporation of fillers like clay, graphene, carbon nanotubes, etc. into the polymer matrix is one of the effective ways to improve the properties of PBS [10,12,13,14,15,16,17,18,19]. For example, the addition of organically modified layered silicate was found to enhance the mechanical properties of PBS composites prepared via melt extrusion [10,14]. In another report, the effect of surface-treated montmorillonite on the thermal and physical properties of PBS was investigated [15]. The results revealed that the modification of the clay helped in getting highly exfoliated nanocomposites with improved mechanical properties. From the literature review, it can be noted that one of the primary objectives of most of the studies on PBS composites is to improve the mechanical properties of PBS.

The addition of certain fillers was also found to improve the degradation of PBS. A study on the influence of sugarcane rind fibre (SRF) on the biodegradability of the PBS/SRF composites under natural soil conditions showed that the incorporation of SRF accelerated the degradation of PBS. The enhanced degradation is due to the hydrolysis of amorphous regions of PBS, which facilitates the activity of microorganisms [20]. The addition of the jute fibre has been found to enhance the biodegradation of PBS in PBS/jute fibre composites buried in compost soil [21]. In another report, an increase in the rate of degradation of PBS was observed with the addition of agro-flour as a filler during a soil burial test [22]. PBS/organo-montmorillonite (OMMT) nanocomposites were found to show lower biodegradability in compost soil, compared to neat PBS. However, the addition of maleic anhydride-grafted PBS (PBS-g-MA) as a compatibilizer had enhanced the biodegradability of the nanocomposites [23].

There are a few reports on the degradation behaviour of PBS under marine conditions [7,24,25,26,27]. In a recent study, biodegradation of different polyesters was investigated in seawater via lab and field tests. PBS was found to exhibit slow biodegradation in the BOD (biochemical oxygen demand) test using seawater. However, minimal weight loss was observed in the field test, in which the specimens were immersed in the sea at Osaka Bay. The difference in degradation behaviour is due to the difference in variety of life in the sea and the physical and chemical effects derived from the environment [24]. In a detailed biodegradation study conducted on a range of selected biodegradable polymers, PBS (trade name: PBE 003) was found to show poor degradation across different tested environments (home composting, anaerobic digestion, marine environment, etc.) [25]. A comparative study on the biodegradability of different aliphatic polyesters such as poly(3-caprolactone) (PCL), poly(β-hydroxybutyrate/valerate) and PBS under marine conditions showed that the deterioration of PBS was minimal compared to the other polymers [27]. In another report, the percentage of biodegradation of pure PBS was found to be very low even after 28 days of immersion in seawater. Moreover, the incorporation of a high amount of PBS was found to inhibit the biodegradability of poly(3-hydroxybutyrate-co-3-hydroxyhexanoate) [26]. From the literature, it is evident that PBS exhibits poor degradation under marine conditions. Therefore, in this work, we focused on understanding the role of fillers to enhance the degradation of PBS under marine conditions. Layered double hydroxide (LDH) is used as the reinforcing filler for making PBS composites due to their ability to catalyse the degradation of polyesters and its low toxicity to the marine environment [28,29,30,31].

Layered double hydroxides (LDHs) are anionic clays, which contain positively charged brucite-like layers with interlayer charge compensating anions and solvation molecules [32]. LDHs can be represented by the general formula [M^2+^_1−x_M^3+^_x_(OH)_2_][A^n−^]_x/n_·zH_2_O, where M^2+^ and M^3+^ are divalent and trivalent metal cations [32]. LDHs are also considered a useful filler to improve the properties of different polymers [33,34,35]. LDHs with different divalent and trivalent metal ions such as Mg-Al LDH and Zn-Al LDH have been used to prepare polymer composites with improved properties [36,37,38,39]. The improvement in the properties of the polymer has a strong dependence on the degree of exfoliation of LDH in the polymer matrix. However, the stronger interlayer interactions in the LDH due to the higher charge density impede efficient exfoliation. Hence, it is always a challenge to prepare highly exfoliated polymer nanocomposites using LDH. To overcome this challenge, LDH is often modified with organic compounds or surfactants to improve its compatibility with the polymer [34,40,41]. There are a few reports on PBS/LDH composites, mainly focusing on improving the mechanical properties of PBS [29,42,43,44,45]. However, there was not much emphasis on the marine biodegradation behaviour of PBS in the presence of LDH.

The focus of the present work is to understand the degradation behaviour of PBS composites under marine conditions in the presence LDH additives. This work has great significance since the accumulation of plastic wastes in the oceans has emerged as a global challenge and only limited experimental data is available on the degradation of plastics in the aquatic environment [1,4]. Herein, we investigated the influence of LDH on biodegradation, along with the mechanical and thermal properties of PBS. Since the degree of dispersion of the filler has a strong influence on the properties of the polymer, we used stearate modified magnesium-aluminium LDH (St-Mg-Al LDH) to make the PBS composites to improve the miscibility of the filler with the polymer matrix.

## 2. Results and Discussion

PBS composites with 2.5, 5 and 10 wt% of St-Mg-Al LDH (hereafter denoted as PBS/2.5 St-Mg-Al LDH, PBS/5 St-Mg-Al LDH and PBS/10 St-Mg-Al LDH, respectively), were prepared using melt-mixing process. The influence of different loadings of St-Mg-Al LDH on different properties of PBS was investigated in detail with the help of various characterisation techniques. The results are discussed below.

### 2.1. Dynamic Mechanical Analysis (DMA)

**Figure 1:**
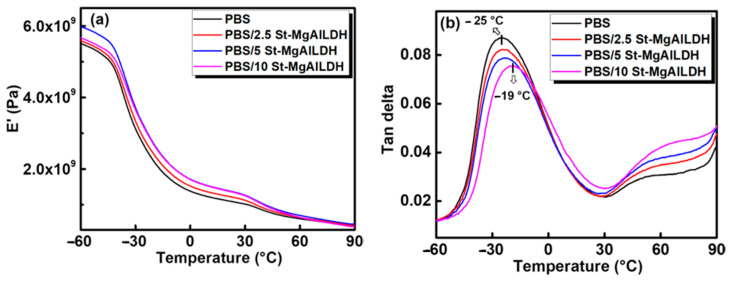
Dynamic mechanical analysis of PBS and PBS/ St-Mg-Al LDH composites: (**a**) storage modulus (E′) and (**b**) tan δ.

The viscoelastic properties of the composites as a function of temperature were investigated using a dynamic mechanical analyser. The results obtained for PBS and PBS composites with different St-Mg-Al LDH loadings are shown in Figure 1. The variations in the storage modulus (E′) and loss tangent (tan δ) will help in understanding the extent of interfacial interaction between the filler and the polymer.

All the PBS/St-Mg-Al LDH composites, especially with 5 wt% St-Mg-Al LDH, showed better E′ compared to pure PBS and a clear difference was observed until 30 °C. At −60 °C, the storage modulus of pure PBS was 5.5 GPa, whereas for PBS/5St-Mg-Al LDH it was 6 GPa. E′ values generally represent the elastic properties of the polymer and are highly sensitive to the dispersion of the filler in the polymer matrix and the interfacial interaction between the phases [46,47]. The enhancement in the E′ values indicates an improvement in the stiffness of PBS [46,47]. A sudden drop in the E′ was observed around −30 °C for all the samples, which can be related to the glass transition temperature (T_g_) of PBS. Therefore, the decrease in the E′ is due to the molecular relaxation at higher temperatures above T_g_ [46]. The T_g_ value of PBS, obtained from the position of tan δ peak maximum (Figure 1b), was found to increase with the addition of St-Mg-Al LDH and became more evident in the case of PBS/10 St-Mg-Al LDH, with an increase of ~6 °C. The incorporation of the fillers can restrict the segmental mobility of the polymer chain, which in turn increases the T_g_ of the polymer [16,44]. With the help of tan δ curves, an increase in the T_g_ of PBS was reported with the addition of both Zn-Al and Mg-Al LDH, which was attributed to the restricted chain mobility of PBS [44]. The incorporation of organically modified clay was also found to increase the T_g_ value of PBS, obtained from the tan δ curve, due to the constrained molecular motion [16]. Therefore, the increase in the T_g_ can be attributed to the limited chain mobility of PBS due to the presence of St-Mg-Al LDH. Moreover, the height of the tan δ peak was found to be less for all the composites compared to PBS. The decrease in the tan δ height again indicates the stiffness improvement of the composites, since the filler can reduce the strain at the interface [47,48,49].

### 2.2. Mechanical Properties

Tensile measurements were carried out to understand the effect of St-Mg-Al LDH on the mechanical properties of PBS. In order to get a proper reinforcement effect, there should be an appropriate transfer of stress from the filler to the polymer matrix and vice versa, which depends entirely on the interaction between the polymer and the filler. Therefore, the mechanical properties of the composites can vary depending on the filler-matrix interactions.

Table 1 represents the variation in the tensile properties of PBS with the addition of St-Mg-Al LDH. Stress vs. strain curves obtained for PBS and PBS/St-Mg-Al LDH are given in the Supplementary Materials (Figure S1). A slight decrease in the tensile strength was observed with the addition of St-Mg-Al LDH. However, there was no significant variation in Young’s modulus. A similar observation was made in another study, where a slight decrease in the tensile strength was observed for PBS/layered silicate nanocomposites, prepared using a melt extrusion process with an increase in the modulus. The decrease in the tensile strength was attributed to the weak interaction between the filler and polymer matrix [11]. Similarly, a significant reduction in the tensile strength was observed for PBS/miscanthus composites compared to PBS. The reduction was correlated with the lack of interfacial interaction and incompatibility between the filler and the polymer. Moreover, similar to the observation made in the present study, an increase in the E′ value was observed for the composites with the addition of miscanthus fibres up to 50 wt% [46]. Bio-PBS/grape pomace (GP) composites were also reported to exhibit similar behaviour, where an increase in the E′ value with a substantial decrease in the tensile strength was observed with the addition of GP [47]. In another report, a substantial decrease in the tensile strength was observed for melt blended PBS/rice husk flour (RHF) composites, prepared using a twin-screw extruder with the increase in the RHF loading. The decrease was correlated to the weak interfacial adhesion between RHF and the PBS matrix, which may lead to the formation of micro-cracks at the interface [22]. Therefore, in the present study, we speculate that the decrease in the tensile strength might be due to the weak interaction of the filler with the polymer matrix [11,12]. The addition of St-Mg-Al LDH has decreased the ductility of PBS, which is evident from the decrease in the elongation at break. This result is in good agreement with the observation made from the DMA, where the decrease in the height of tan δ peak for the composites indicated an increase in the stiffness. Moreover, the toughness, calculated by integrating the area under the stress-strain curve, was found to decrease with the filler addition. This observation again indicates a weak interaction between the filler and the polymer matrix [50,51].

By analysing the tensile and dynamic mechanical properties, it appears that the dispersion of LDH is not adequate in the PBS matrix to improve the mechanical properties. A similar observation was made in another study, where the thermal, rheological and dynamic mechanical properties of PBS/organo-modified LDH composites, prepared via melt compounding and in-situ polymerisation, were investigated [29]. From the results, only minor changes in the crystallisation rate and no enhancement in the dynamic mechanical properties were observed for the melt compounded samples. However, improved mechanical properties were reported for the samples prepared using an in-situ polymerisation method due to improved dispersion and delamination of LDH filler in the PBS matrix. However, in the present study, it should be noted that there was no significant deterioration of the tensile properties of PBS, but at the same time, an enhancement in the storage modulus was observed for PBS/St-Mg-Al LDH composites, compared to neat PBS.

### 2.3. Crystallization Behaviour

**Figure 2:**
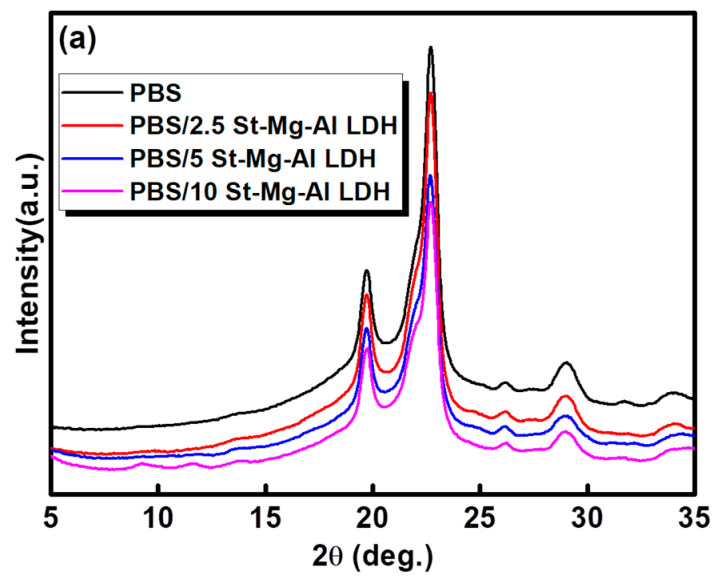

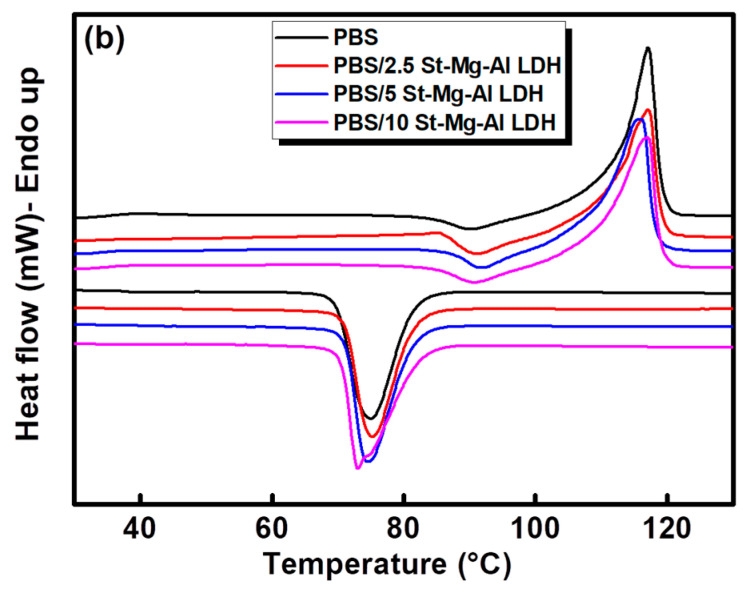
(**a**) XRD patterns and (**b**) DSC first heating and first cooling (non-isothermal melt crystallization) curves obtained for PBS and PBS/St-Mg-Al LDH composites.

XRD measurements were carried out to understand the changes in the crystalline structure of PBS with the addition of St-Mg-Al LDH. Figure 2a represents the XRD patterns obtained for PBS and PBS/St-Mg-Al LDH composites. The XRD patterns showed the typical semicrystalline nature of PBS and the peaks were observed at 2θ = 19.7°, 22.1°, 22.7°, 26.2° and 29.1° [52,53,54]. No major changes in the crystal structure were observed after the addition of St-Mg-Al LDH. The percentage of crystallinity was also calculated from the XRD diagram (see Table S1 in Supplementary Materials). It was observed that the incorporation of St-Mg-Al LDH could not induce any major changes in the crystallinity of PBS.

DSC measurements were carried out to understand the changes in the crystallization behaviour of PBS with the addition of St-Mg-Al LDH. DSC first heating and first cooling (non-isothermal melt crystallization) curves obtained for the PBS and PBS/St-Mg-Al LDH composites at the rate of 10 °C/min are compared in Figure 2b. The addition of the filler had almost no influence on the melting and melt crystallization behaviour of PBS. The results imply that the LDH could not act as an effective heterogeneous nucleating agent to facilitate the crystallization rate of PBS.

### 2.4. Thermal Stability

**Figure 3:**
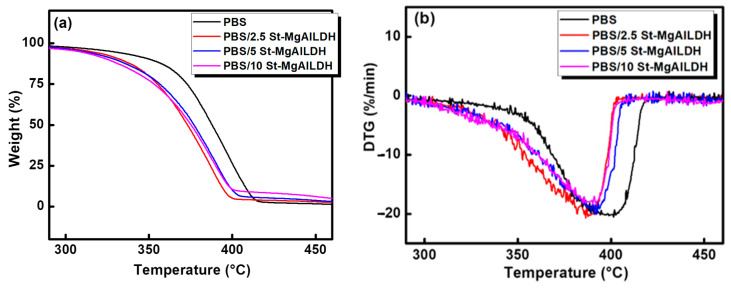
(**a**) TGA and (**b**) DTG curves obtained for PBS and PBS/St-Mg-Al LDH.

TGA analysis was performed to understand the effect of different loadings of St-Mg-Al LDH on the thermal stability of PBS. The TGA and DTG thermograms obtained for PBS/St-Mg-Al LDH composites are shown in Figure 3. It is evident from the figure that the presence of St-Mg-Al LDH has reduced the thermal stability of PBS irrespective of filler loading. For all the samples, the prime decomposition was found to be in the temperature range 300–450 °C.

Table 2 compares the onset degradation temperature (T_onset_) and variation in the 50% weight loss temperature (T_0.5_) with the addition of St-Mg-Al LDH. A decrease of 14 °C in the onset of degradation (T_onset_) and 13 °C decrease in the 50% weight loss temperature (T_0.5_) value was observed with the addition of 10% St-Mg-Al LDH (Table 2).

It is reported that the Mg and Al ions in the LDH can catalyse the transesterifications of the polyesters and accelerate the thermal degradation [28,29,30]. The presence of Mg and Al, the metal constituents of LDH, was found to accelerate the chain hydrolysis and catalyse the intermolecular and intramolecular transesterifications of PBS in PBS/LDH nanocomposites. Thiscould be resulted in the decreased thermal stability of PBS in the nanocomposites [30]. The incorporation of organically-modified magnesium/aluminium layered double hydroxide (P-LDH) was also found to reduce the thermal stability of poly(l-lactide) (PLLA) due to the depolymerisation reaction, catalysed by the Mg and/or Al components in P-LDH [28]. Therefore, in the present study, the decrease in the thermal stability with the addition of St-Mg-Al LDH might be due to the depolymerisation of PBS caused by the catalytic action of Mg and/or Al.

### 2.5. Biodegradation under Marine Conditions

**Figure 4:**
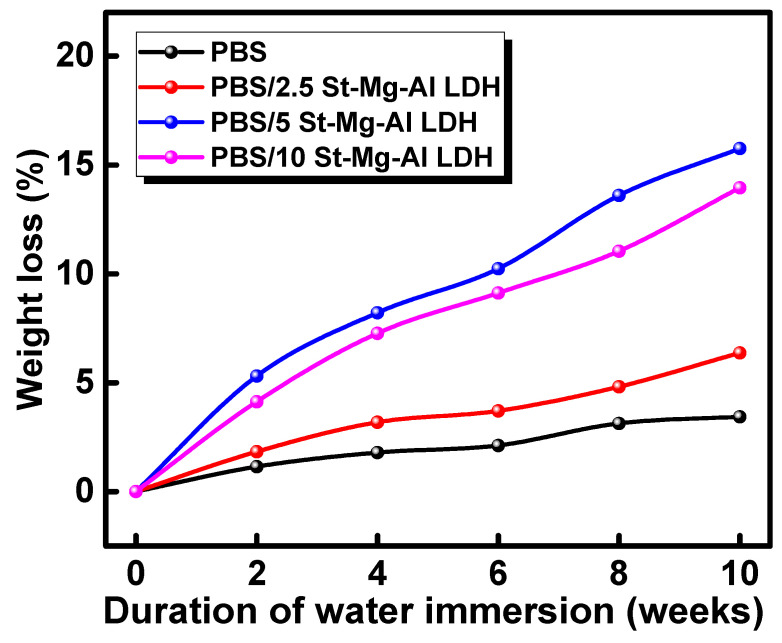
Percentage of weight loss obtained for PBS/St-Mg-Al LDH composites with time.

PBS composites films with a thickness of ~0.01 cm were immersed in seawater, and their degradation behaviour was analysed for 70 days under simulated conditions. The assessment of the degradation was based on the weight loss and surface morphology analysis performed at particular time intervals. Figure 4 represents the weight loss of the seawater-immersed composite film with time.

It is evident from Figure 4 that the percentage of weight loss has increased with time for all the samples. However, a significant increase in weight loss was observed for PBS/St-Mg-Al LDH composites compared to pure PBS. Among the composites, the maximum weight loss was observed for PBS/5 St-Mg-Al LDH composites. The results indicate that the incorporation of St-Mg-Al LDH has accelerated the degradation of PBS under marine conditions. In a previous report on the biodegradability of SRF/PBS composites under natural soil conditions, a maximum weight loss of ~19.2% was observed for the composites with 5 wt% SRF loading after soil burial for 100 days [20]. However, from previous reports, it was understood that the degradation of PBS is slow in seawater [7,24,25,26,27]. A recent investigation on the biodegradation of synthetic polyesters immersed in seawater showed that the weight loss of PBS was minimal (below 5%) even after 6 weeks of seawater immersion [24]. On the other hand, in the present study, we could observe a weight loss of ~10% after 6 weeks and a maximum weight loss of ~16% for the PBS/5 St-Mg-Al LDH composite after ten weeks (70 days) of seawater immersion.

**Figure 5:**
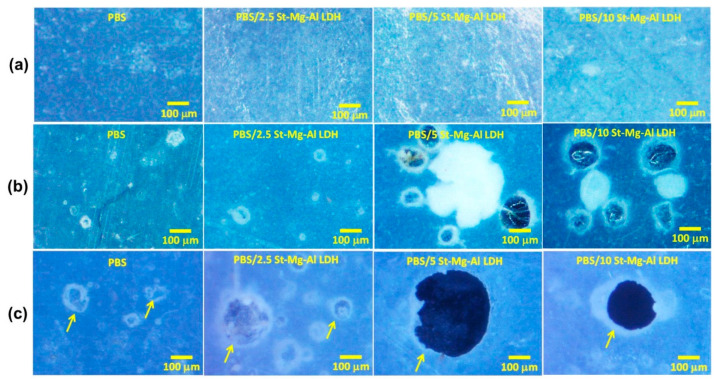
Optical images captured for PBS/St-Mg-Al LDH composites: (**a**) before seawater immersion, (**b**) after 5 weeks and (**c**) 10 weeks of seawater immersion.

Optical imaging of the composite films was performed at different time intervals to see the changes in their surface with time. The optical images obtained before and after 5 and 10 weeks of seawater immersion are shown in Figure 5.

The optical images (Figure 5) clearly show pore formation (highlighted with arrows) in all the samples with time. It is evident from Figure 5 that the rate of degradation is quite slow for the neat PBS film. However, the addition of St-Mg-Al LDH has significantly improved the surface erosion of PBS. After 5 weeks of seawater immersion, the surface degradation was very much evident in the composite films, especially with 5 and 10 wt% St-Mg-Al LDH loadings. A substantial improvement in the degradation can be observed after 10 weeks of seawater immersion, and the optimum result was obtained for PBS/5 St-Mg-Al LDH composite. SEM measurements were carried out to gather more information about the changes in the surface morphology of the composite films which are immersed in seawater.

**Figure 6:**
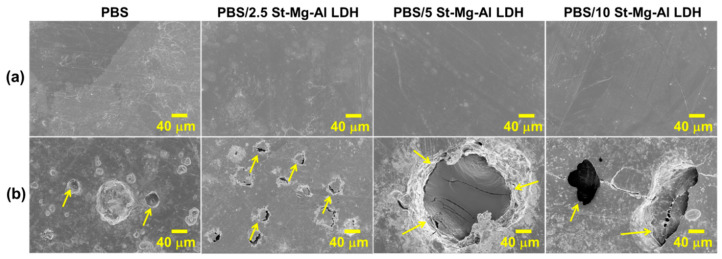
SEM images obtained for PBS/St-Mg Al LDH composites: (**a**) before seawater immersion, (**b**) after 10 weeks of seawater immersion.

Figure 6 shows the SEM images obtained for the PBS composites before and after 10 weeks of seawater immersion. The SEM analysis gives more evidence for the surface degradation of PBS composites (highlighted with arrows). Similar to the observation made from the optical imaging, the addition of 5 and 10 wt% St-Mg-Al LDH loadings were found to enhance surface erosion of PBS compared to the other composites. Average void sizes of PBS and PBS/St-Mg-Al LDH composites, calculated from the SEM images, are shown in Figure S2 (Supplementary Materials). A significant increase in the void size was observed for the PBS composites compared to pure PBS, and the maximum increase was observed for PBS/5 St-Mg-Al LDH composite. Therefore, it is evident that the addition of LDH is playing a crucial role in the degradation of PBS. The inferior degradation of PBS/10 St-Mg-Al LDH compared to PBS/5St-Mg-Al LDH might be due to the aggregation of the filler at 10% loading. Therefore, in the present study, 5% filler loading can be considered as the optimal filler loading.

**Figure 7:**
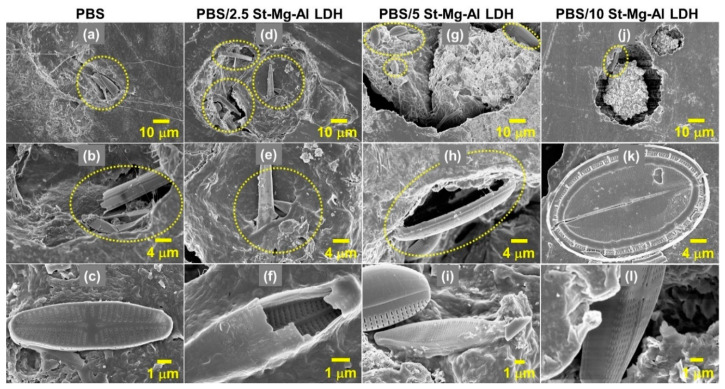
SEM images, which show the presence of microorganisms (diatoms), obtained for PBS composites after 10 weeks of seawater immersion; PBS (**a**–**c**), PBS/2.5 St-Mg Al LDH (**d**–**f**), PBS/5 St-Mg Al LDH (**g**–**i**) and PBS/10 St-Mg Al LDH (**j**–**l**).

Apart from the formation of voids, the attachment of microorganisms to the surface of PBS composites is evident in the SEM images, as shown in Figure 7. The structures shown in Figure 7 were similar to the reported SEM images of diatoms [55,56]. Diatoms are unicellular, eukaryotic microorganisms with cell walls heavily impregnated with silica [57,58]. SEM images substantiate the colonisation of different kinds of diatoms on the surface of seawater-immersed polymer films, especially near the degraded area (Figure 7).

**Figure 8:**
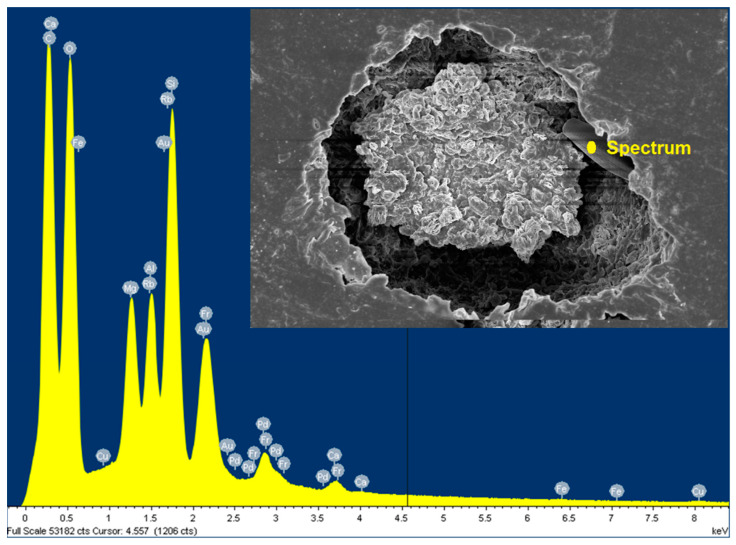
SEM energy dispersive X-ray analysis (SEM-EDX) analysis of PBS/St-Mg Al LDH after 10 weeks of seawater immersion.

SEM energy dispersive X-ray analysis (SEM-EDX) was performed to identify the elements present near the degraded area in the PBS/St-Mg Al LDH composite after the seawater immersion. The results (Figure 8) clearly show the presence of Mg and Al, which proves the elemental composition in the LDH. Moreover, the detection of silicon (Si) in the EDX spectrum further confirms the presence of diatoms, since their cell wall is heavily impregnated with silica. It is evident from Figure 8 that the presence of Mg, Al and Si was detected near the degraded area.

Any surface immersed in seawater is susceptible to biofouling (accumulation of microorganisms)/biofilm formation and biodegradation [59,60,61]. Similarly, when a polymer film is exposed to seawater, the accumulation of microorganisms leads to the formation of biofilm [60,62]. The formation of biofilm provides a proper platform for the settlement of organisms such as microalgae (including diatoms) and microscopic fungi [63,64], which can influence the biodegradation of the polymer via surface erosion [65]. Microbial colonisation and biofilm formation in the plastic marine debris (PMD) was examined with the help of SEM analysis [66]. The results revealed that microbial communities hydrolyse the PMD and accelerate the physical degradation of plastics [66]. Similarly, in another report, the presence of varieties of bacteria and algae on the edges and surface of poly(3-hydroxybutyrate-co-3-hydroxyhexanoate) samples was observed after the seawater treatment. The void formation observed for the samples after the seawater exposure was correlated with the high microbial activity on the surface of composite samples [67]. Pelegrini et al. investigated the degradation behaviour of poly(lactic acid) (PLA) composites in a simulated marine environment. The results showed that the colonisation of microorganisms (diatoms) has a strong influence on the degradation of PLA composites [68].

In the present study, the SEM analysis confirms the presence of diatoms in the vicinity of the degraded areas of the polymer film. Therefore, the microbial activity by the colonisation of microorganisms might be one of the reasons for the degradation of the PBS composite films in the presence of LDH. Previous reports suggest that the degradation of PBS is poor under marine conditions [7,24,25,26,27]. However, in the present study, the addition of LDH has played a crucial role in enhancing the degradation of PBS along with the microbial activity.

The presence of nanoclay is reported to accelerate the hydrolytic degradation of the polymer matrix due to the presence of hydroxyl groups on the edges of the clay [69,70,71,72]. The addition of organically modified layered silicate clay was found to enhance the hydrolysis and controls the degradation of PLA [71,72]. The improved hydrolysis was correlated with the presence of terminal hydroxylated edge groups of the silicate layers. The biodegradability of PBS was also found to improve after the incorporation of layered silicate clay [11]. The nature of clay modifier can also influence the biodegradation of PBS [73]. To the best of our knowledge, there are no reports on the effect of LDH on the biodegradation of PBS under marine conditions. Similar to the degradation mechanism reported for polymer/nanoclay composites, in the present study we speculate that the catalytic activity of St-Mg-Al LDH might have accelerated the hydrolytic degradation of PBS due to the presence of surface hydroxyl groups in LDH. Moreover, an improvement in the biodegradation of PLA was reported due to the faster degradation rate of stearate groups in PLA/stearate-Zn_3_Al LDH nanocomposites, which causes the breakdown of PLA matrix into smaller particles [74]. Therefore, the degradation of stearate groups might have also helped in accelerating the degradation of PBS in PBS/St-Mg-Al LDH composites. However, a more detailed investigation is required to better understand the degradation mechanism of PBS in the presence of LDH.

## 3. Materials and Methods

### 3.1. Materials

PBS (pellets) (commercial name: PBE003) was purchased from Natureplast company (Ifs, France). Stearate modified magnesium-aluminium layered double hydroxide (St-Mg-Al LDH) under the commercial name “Hydrotalcite MgAl-hydroxide stearate”was purchased from Prolabin & Tefarm manufacturer (Perugia, Italy).

### 3.2. Preparation of Polymer Composites

Polymer composites were prepared via a melt-mixing process using a lab-scale Brabender. Both polymer and LDH samples were dried under vacuum at 80 °C overnight. The samples were mixed with a rotor speed of 50 rpm at 150 °C for 10 min. PBS composites with 2.5, 5 and 10 wt% of St-Mg-Al LDH were prepared, hereafter denoted as PBS/2.5 St-Mg-Al LDH, PBS/5 St-Mg-Al LDH and PBS/10 St-Mg-Al LDH, respectively. The melt-processed samples were compression moulded to rectangular specimens with thicknesses of 0.1 and 0.01 cm at 150 °C under 200 bar pressure using a Servitech Polystat 200 T compression press (Wustermark, Germany) for further analysis and characterisation.

### 3.3. Characterisation

#### 3.3.1. Biodegradation in Seawater

The degradation behaviour of the PBS composites in seawater was studied using the films with dimensions 2 × 2 × 0.01 cm. The samples were placed in bottles filled with water collected from the Irish Sea/Liffey River, Dublin, and the bottles were placed on a rocking plate to create a vortex of water to keep the maximum contact with the plastic samples. The temperature of the water was maintained at ~20–22 °C throughout the experiments and the pH was measured to be ~8.2–8.3. The water was replaced every seven days, and the volume of the water was maintained at around 70 mL. The experimental setup used for the degradation study is shown in Supplementary Materials (Figure S3).

#### 3.3.2. Weight Loss Analysis

Each specimen was taken out periodically (every two weeks) from the seawater and dried to a constant weight at 60 °C in a vacuum oven. Table S2 in the supporting information shows the variation in the weight obtained for different samples with time. The percentage of weight loss was measured using a high precision electronic balance and calculated using Equation (1).

(1) $$\mathrm{Weight}\;\mathrm{loss}=\frac{W_{\mathrm{initial}}\;-W_{\mathrm{final}}}{W_{\mathrm{initial}}}\times 100\;\%$$

where W_initial_ and W_final_ is the weight of the sample before and after the seawater immersion.

#### 3.3.3. Tensile Testing

Tensile measurements were performed using a Zwick twin column tensile tester (ZwickRoell, Kennesaw, GA, USA) with a 2.5 kN load cell. Dumbbell-shaped specimens of PBS composites with dimensions of 75 mm × 4 mm × 1 mm were used for the analysis. The tensile tests were carried out at a crosshead speed of 50 mm/min at room temperature.

#### 3.3.4. Dynamic Mechanical Analysis (DMA)

The dynamic mechanical properties of the PBS composites were analysed using a PerkinElmer Diamond DMA (PerkinElmer, Waltham, MA, USA). Samples with dimensions 20 mm × 4 mm × 1 mm were heated from −60 °C to 110 °C with a purging liquid N_2_. A heating rate of 2 °C/min was used to be slow enough to thermally equilibrate each specimen in the furnace. The composite specimen was deformed in a tension mode at a fixed frequency of 1 Hz. The oscillation amplitude used was 0.2 mm. The tests were performed under a nitrogen atmosphere.

#### 3.3.5. Thermogravimetric Analysis (TGA)

The thermal stability of PBS composites was examined using a PerkinElmer Pyris 1 TGA (PerkinElmer). Each sample (ca. 3 mg) was loaded in a platinum pan and heated from 30 °C to 700 °C at the rate of 10 °C/min under air atmosphere.

#### 3.3.6. Differential Scanning Calorimetry (DSC)

A PerkinElmer Pyris Diamond Differential Scanning Calorimeter (DSC) (PerkinElmer) was used to understand the crystallization behaviour of the PBS composites. The machine was calibrated using indium standards. The samples (~3–5 mg) were sealed in aluminium pans and heated from −60 to 140 °C at a rate of 10 °C/min and held at 140° C for 2 min to erase the thermal history. Then, the melt was cooled at a rate of 10 °C/min to understand the non-isothermal melt crystallization behaviour.

#### 3.3.7. X-Ray Diffraction (XRD) Measurements

XRD measurements were carried out on a XEUSS SAXS/WAXS system (Xenocs, Grenoble, France) (operated at 50 kV and 0.6 mA). The X-ray beam was collimated with a FOX2D mirror and two pairs of scatterless slits. The fibre diagrams were recorded on a Mar345 image plate and processed using Fit2D software (Version: V12.081, Andy Hammersley/ ESRF, Grenoble, France). All the measurements were performed in the transmission mode. The following equation was used to calculate the degree of crystallinity (*X_c_*) based on the scattering intensities of crystalline (*A_c_*) and amorphous (*A_a_*) phases.

(2) Xc= AcAc+Aa 

#### 3.3.8. Optical Imaging

The optical microscope imaging was performed using a Zeiss Stemi (Version:2000-C, Carl Zeiss, Oberkochen, Germany) stereo microscope, and the images were captured using a Zeiss Axiocam MRc digital camera (Carl Zeiss) attached to the microscope.

#### 3.3.9. Scanning Electron Microscopy (SEM)/Energy Dispersive X-ray Spectroscopy (EDX)

The surface morphology of PBS composites was analysed using a high-resolution field emission Zeiss Ultra Plus-SEM (Carl Zeiss), which operates with an accelerating voltage of 5 kV. The samples were pasted onto the SEM stubs using carbon tape and sputtered with gold/palladium (80/20 ratio) for 15 s. EDX analysis, which is an integral characteristic of the Zeiss Ultra Plus-SEM, was used to identify the elemental composition of the sample. Point scans were performed at different areas at a working distance of 10 mm with the Energy selective Backscattered (EsB)detector.

## 4. Conclusions

Herein, we report the enhanced biodegradation of PBS in the presence of St-Mg-Al LDH under simulated marine conditions. The degradation of the seawater-exposed composite films was confirmed with the help of optical imaging, SEM and weight loss analysis. The results revealed that the addition of St-Mg-Al LDH enhanced the degradation PBS and the optimum results were obtained for the composites with 5 wt% St-Mg-Al LDH loading. It is speculated that the catalytic effect of St-Mg-Al LDH might be one of the primary reasons for the degradation of PBS in seawater. Moreover, the SEM analysis disclosed that seawater exposure has resulted in the colonization of microorganisms on the surface of the polymer films. The microbial activity of such adhered microorganisms and the presence of LDH also play a vital role in accelerating the degradation of PBS. The incorporation of St-Mg-Al LDH was found to decrease the thermal stability of PBS. It was correlated to the enhanced degradation of PBS due to the transesterification reaction caused by the catalytic action of Mg and/or Al. On the other hand, the mechanical properties of PBS were not found to change significantly with the incorporation of St-Mg-Al LDH.

## Figures and Tables

**Table 1:** Tensile properties of PBS/St-Mg-Al LDH composites measured at room temperature (~22 °C).

Samples	Tensile Strength (MPa)	Young’s Modulus (GPa)	Elongation at Break (%)	Toughness (MJ/m^3^)
PBS	32.6 ± 1.7	0.41 ± 0.005	10.5 ± 1.2	2.21 ± 0.48
PBS/2.5 St-Mg-Al LDH	29.6 ± 0.2	0.42 ± 0.0003	9.5 ± 0.06	1.80 ± 0.07
PBS/5 St-Mg-Al LDH	27.8 ± 1.2	0.45 ± 0.01	8.3 ± 0.3	1.47 ± 0.17
PBS/10 St-Mg-Al LDH	24.8 ± 0.5	0.47 ± 0.02	7.5 ± 0.08	1.28 ± 0.10

**Table 2:** Thermal stability of PBS/St-Mg-Al LDH composites.

Samples	The Onset of Degradation (T_onset_)	The 50% Weight Loss Temperature (T_0.5_)
PBS	363.4 °C	387.7 °C
PBS/2.5 St-Mg-Al LDH	348.3 °C	373.1 °C
PBS/5 St-Mg-Al LDH	352.6 °C	377.0 °C
PBS/10 St-Mg-Al LDH	349.4 °C	375.1 °C

## Supplementary Materials

The following are available online, Figure S1: Stress vs. strain curves of PBS and PBS/St-Mg-Al LDH composites, Figure S2: The average pore sizes of PBS and PBS/St-Mg-Al LDH composites calculated from SEM, Figure S3: The experimental setup used for the degradation study of PBS composite films in water, Table S1: Variation in the percentage of crystallinity obtained for pure PBS and PBS composites, Table S2: Variation in the weight obtained for pure PBS and PBS composites after seawater immersion.
