# Supplementary material for: Biodegradation of Poly (Butylene Succinate) (PBS)/Stearate Modified Magnesium-Aluminium Layered Double Hydroxide Composites under Marine Conditions Prepared via Melt Compounding
Source: Molecules. 2020 Dec 7;25(23):5766. doi: 10.3390/molecules25235766 (PMC7730599; doi:10.3390/molecules25235766)
Supplement: Supplementary file 1 [file molecules-25-05766-s001.pdf]

SUPPORTING INFORMATION

Biodegradation of Poly (Butylene Succinate) (PBS)/ Stearate Modified Magnesium-Aluminium Layered Double Hydroxide Composites under Marine Conditions Prepared via Melt Compounding

Parameswaran Shaiju [1], Benamor-Bois Dorian [2], Ramsankar Senthamaraikannan [2] and Ramesh Babu Padamati [1,2,*]

[1] BiOrbic, Bioeconomy Research Centre, University College Dublin, Belfield, Dublin D04 V1W8, Ireland; sparames@tcd.ie

[2] AMBER Centre, CRANN Institute, School of Physics, Trinity College Dublin, Dublin D02 PN40, Ireland; d.benamorbois@gmail.com (B.-B.D.); ramsankar.s@tcd.ie (R.S.)

* Correspondence: babup@tcd.ie; Tel.: +353-18962602

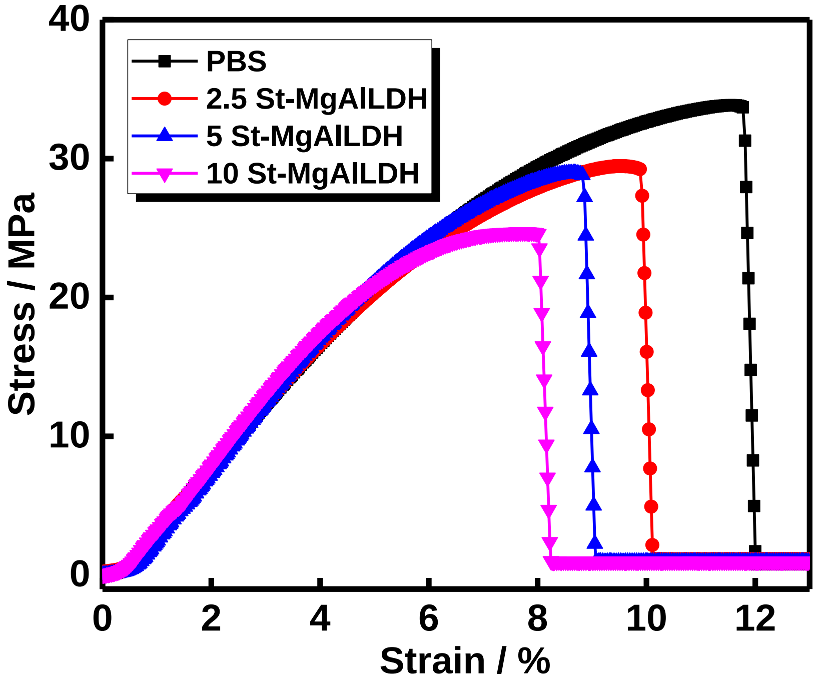

Figure S1. Stress vs. strain curves of PBS and PBS/ St-Mg-Al LDH composites.

Table S1. Variation in the percentage of crystallinity obtained for pure PBS and PBS composites.

Samples	Crystallinity (%)
PBS	48.0
PBS/2.5 St-Mg-Al LDH	48.5
PBS/5 St-Mg-Al LDH	47.5
PBS/10 St-Mg-Al LDH	48.7

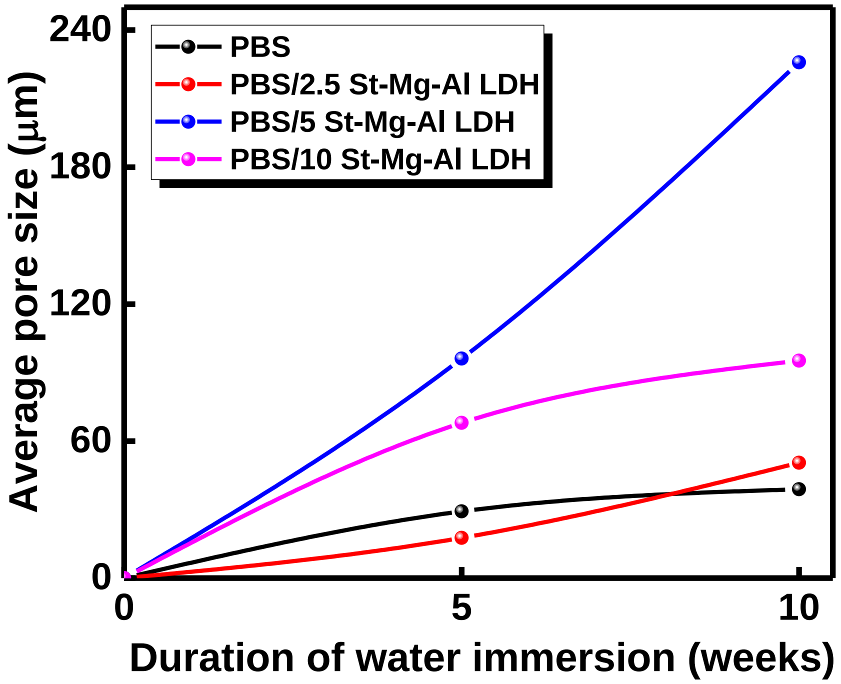

Figure S2. The average pore sizes of PBS and PBS/ St-Mg-Al LDH composites calculated from SEM.

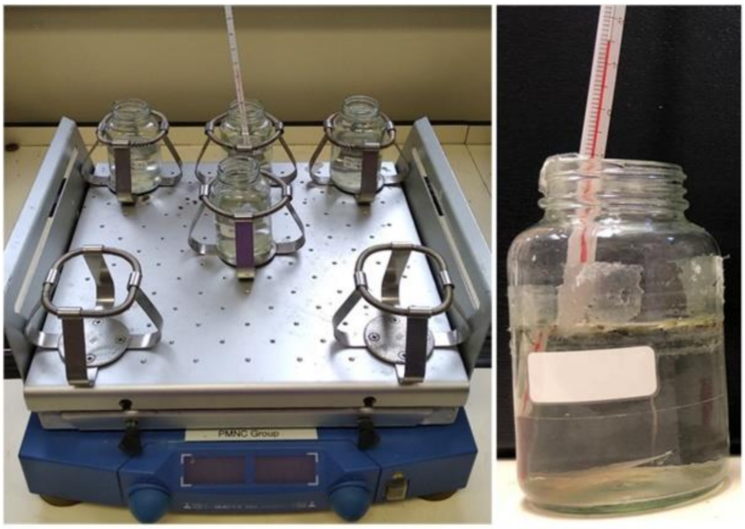

Figure S3. The experimental setup used for the degradation study of PBS composite films in water.

Table S2. Variation in the weight obtained for pure PBS and PBS composites after seawater immersion.

Duration of water immersion	Weight of the sample (mg)			
	PBS	PBS/2.5 St-MgAlLDH	PBS/5 St-MgAlLDH	PBS/10 St-MgAlLDH
0 weeks	52.4	53.4	50.9	50.9
2 weeks	51.8	52.42	48.2	48.8
4 weeks	51.46	51.7	46.72	47.2
6 weeks	51.29	51.42	45.69	46.26
8 weeks	50.76	50.83	43.98	45.28
10 weeks	50.6	50	42.88	43.8
